# Letter to editor on “Thirty‐six‐month results of laparoscopic‐based renal denervation plus unilateral laparoscopic adrenalectomy for the treatment of patients with resistant hypertension caused by unilateral aldosterone‐producing Adenoma”

**DOI:** 10.1111/jch.14416

**Published:** 2021-12-29

**Authors:** Donatella Schiavone, Maurizio Iacobone, Gian Paolo Rossi

**Affiliations:** ^1^ PhD Arterial Hypertension and Vascular Biology Department of Medicine – DIMED University of Padua Padova Veneto Italy; ^2^ Endocrine Surgery Unit Department of Surgery Oncology and Gastroenterology University of Padua Padova Veneto Italy; ^3^ Hypertension and Emergency Unit Department of Medicine – DIMED University of Padua Padova Veneto Italy

Liu and colleagues^1^ investigated the long‐term clinical results of unilateral laparoscopic adrenalectomy (LA) and a renal artery denervation (LA‐RDN) performed from the adventitia in selected patients with resistant hypertension (RH) caused by unilateral aldosterone‐producing adenoma (APA).

By this complex procedure, they claimed to have achieved sustained lowering of blood pressure (BP) and resolution of RH at three years follow up with LA‐RDN. Moreover, owing to the lack of any peri‐procedural complications in their hands, they suggested that this procedure would be safe. However, in our view, their study was affected by several issues that question the validity of these conclusions.

First, the authors stated that they used the American Heart Association definition of RH^2^ as the main inclusion criteria. However, from the mean number of drugs ± SD (3.57 ± .69 and 3.39 ± .50 in the LA‐RDN and control group, respectively, shown in Table 3), it can be calculated that the 95% CI were 2.19–4.95 and 2.39–4.39, indicating that in both groups, they enrolled patients that were on less than three antihypertensive drugs/day. Hence, the fact that not all patients of theirs had RH, indicates an obvious inclusion bias that is relevant for their conclusions. Moreover, it should be noted that the authors reported neither the defined daily dose (DDD), nor the duration of the hypertension and the concurrence of hypertension‐mediated organ damage, all factors which could influence the primary endpoint of their study, besides the patients’ outcome. The authors claimed the superiority of LA‐RDN over LA alone based on a decrease in the number of anti‐hypertensive medications. However, they should have reported the concomitant changes in DDD, since patients can require less drugs but higher doses of each drug to control their high BP values.

Second, the authors stated that their definition of cure and remission of RH differed from that of the PASO study.^3^ For this reason, it would be important to describe in detail what definition of clinical cure, improvement and failure, they used what were the biochemical outcomes for the entire follow‐up period. This is because adrenal venous sampling (AVS)‐guided unilateral LA is itself a safe procedure that leads to biochemical cure in over 95% of the patients and was reported to resolve RH in all APA patients in experienced hands.^4^

Finally, unless the authors postulated that activation of the sympathetic nervous system contribute to causing RH in PA, the rational for undertaking this study is uncertain. In truth, they did not furnish any evidence for this sympathetic activation contribution to RH, while available studies did not support the occurrence of such activation in hyperaldosteronism, thus making unlikely the hypothesis underlying the study by Liu and colleagues.^1^

In summary, there is no question that unilateral LA remains the gold standard for the treatment of patients with AVS‐proven unilateral APA. However, the performance of RDN, be it intraluminal or adventitial, as performed the study by Liu and colleagues,^1^ does not stand on solid pathophysiological premises and should not be performed in these patients, unless they remain resistant to drug treatment after surgery, which, as mentioned above, never was the case in our series of AVS‐guided unilateral APA.^4^

## COMPETING INTEREST

The authors have no competing interests.
